# An improved Diagnostic PCR Assay for identification of Cryptic Heterozygosity for CGG Triplet Repeat Alleles in the Fragile X Gene (FMR1)

**DOI:** 10.1186/1755-8166-1-5

**Published:** 2008-04-08

**Authors:** Mahmoud S Khaniani, Paul Kalitsis, Trent Burgess, Howard R Slater

**Affiliations:** 1 Cyto-Molecular Diagnostics Research Group, Melbourne, Australia; 2 Chromosome and Chromatin Research Group, Murdoch Children's Research Institute, Melbourne, Australia; 3 Department of Paediatrics, University of Melbourne, Victoria, Australia; 4 Tabriz University of Medical Sciences, Tabriz, Iran

## Abstract

**Background:**

Fragile X syndrome (OMIM #300624) is the most common, recognised, heritable cause of mental retardation. Widespread testing is warranted by the relatively high frequency of the disorder, the benefits of early detection and the identification of related carriers whose offspring are at a 1 in 2 risk of inheriting the expanded pathogenic mutation. However, cost-effective screening of mentally retarded individuals has been impeded by the lack of a single, simple laboratory test. Currently, Fragile X syndrome can be excluded in males and a majority of females using a simple high-throughput PCR test. Due to the limited sensitivity of the PCR test, we find in our diagnostic service that approximately 40% of females appear homozygous and a labour intensive and expensive Southern blot test is required to distinguish these from females carrying one normal allele and an expanded allele.

**Results:**

We describe an improved PCR test which displays a high level of precision allowing alleles differing by a single triplet to be resolved. Using the new assay, we detected 46/83 (53%) cryptic heterozygotes previously labelled as homozygotes. The assay also extended the range of repeats amplifiable, up to 170 CGG repeats in males and 130 CGG repeats in females. Combined with the high precision, the assay also improves discrimination of normal (CGG repeats < 45) from grey zone (45 < CGG repeats < 54) alleles and grey zone alleles from small premutations (55 < CGG repeats < 100).

**Conclusion:**

Use of this PCR test provides significantly improved precision and amplification of longer alleles. The number of follow-up Southern blot tests required is reduced (up to 50%) with consequent improvement in turnaround time and cost.

## Background

Fragile X syndrome (FXS), (OMIM #300624) is the most common heritable cause of mental retardation, affecting approximately 1 in 3000 males and 1 in 5000 females. As the phenotypic presentation and frequency of clinical signs are variable, clinical diagnosis is challenging and definitive diagnosis in suspected individuals requires molecular measurement of allele size. Selected screening is highly justified among mentally retarded individuals, not only to provide the benefits of early clinical intervention but very importantly, to enable prenatal diagnosis to be offered to related carriers whose offspring are at a 50% risk of inheriting the expanded mutations which cause clinical abnormality. However, no simple cost-effective technical approach has been developed.

It is well established that FXS is caused by the large scale expansion, beyond the normal number, of CGG repeats in the 5'-untranslated region of the Fragile X mental retardation 1 *(FMR1) *gene with consequent hypermethylation of promotor regions and shutdown of gene expression [1]. Recently, several other disorders have been attributed to smaller scale expansions, namely Fragile X Tremor Ataxia syndrome (FXTAS) (OMIM #300623) and Premature Ovarian Failure (POF) (OMIM # 311360) [2]. Precise measurement of CGG triplet expansion is therefore increasingly important, in particular around the grey zone and premutation repeat thresholds. Several polymerase chain reaction (PCR) assays using a variety of primers and enzymes have been developed for diagnostic use with variable results. An assay with very high precision and the capability of identifying larger alleles is required to detect premutations (54 to 200 repeats) and identify homozygous alleles in females. The latter must be further analysed by Southern blotting to exclude the presence of large un-amplifiable mutations. Because most PCR assays for CGG repeats in diagnostic use have imprecision of at least two repeats in the normal allele range, some apparent homozygotes are likely to be cryptic heterozygotes consisting of alleles differing by 1 or 2 repeats. Assays for CGG repeats must overcome the significant problem of amplifying long tracts of repetitive CG-rich sequence and the associated phenomenon of enzyme stuttering which produces heterogeneous products differing in length by 3 to 4 repeats, especially for alleles longer than 50 repeats. To address these issues, we modified our existing PCR method to improve its performance in terms of expanding the range of amplifiable alleles and increasing precision.

## Methods

### Sample collection

We analysed DNA samples with alleles in the normal (n = 35, ≤ 45 CGG repeat) and intermediate ranges (n = 30, 45 to 54 CGG), the premutation range (n = 26, 55 to 200 CGG), and in the full mutation range (n = 15, > 200 CGG). The sizes of the tri-nucleotide repeat in all the samples had previously been determined using our old PCR assay (CGG < 80) or by Southern Blot analysis (CGG > 80) (see below for details).

DNA was obtained from buccal and venous blood samples. Buccal cells were collected by rolling the brush firmly on the inside of each cheek approximately 20 times. Brushes were stored dry at 4°C and then place into a tube containing 400 μl of 50 mM NaOH and rotated a minimum of 5 times. The tubes were incubated at 95°C for 15 minutes and then 80 μl of Tris-HCl pH 7.5 added to each tube and stored at -20°C.

DNA was obtained from venous blood samples anticoagulated with EDTA. DNA was extracted using a BIO ROBOT M48 DNA Extractor (QIAGEN).

### Molecular Analysis

The old PCR was modified from a previously published protocol using primers *A *(5'-GGAACAGCGTTGATCACGTGACGTGGTTTC-3') and *571R *(5'-GGGGCCTGCCCTAGAGCCAAGTACCTTGT-3') [3]. Amplifications were performed in a 25 μl reaction volume containing 2.5 μl dNTPs (2 mM), 0.5 μl Pfu exo (-)enzyme (Strategene) (2.5 U/μl), 2.5 μl Pfu buffer, 3.1 μl DMSO, 0.5 μl of primer *A *(165 ng/μl) and 0.5 μl of primer *571R *(165 ng/μl) and 45–60 ng of genomic DNA. The thermocycling program consisted of 5 minutes denaturation at 98°C, followed by 35 cycles at 98°C for 1 minute, 62°C for 1 minute and 72°C for 2 minutes, final extension of 5 minutes at 72°C in the Gene Amp^@ ^PCR System 9700 (Applied Biosystem). Results were standardised using commercial controls of 23 and 30 repeats (Coriell Cell Repositories) measured by sequencing.

The improved assay used primers:

*c *(5'-GCTCAGCTCCGTTTCGGTTTCACTTCCGGT-3') and *f *(5'AGCCCCGCACTTCCACCACCAGCTCCTCCA-3')(Sigma) [4].

PCR amplifications were performed in a total volume of 25 μl containing 50 ng of genomic DNA or 2 μl of lysate from buccal swabs, 0.75 pmol of each primer, 8 μl of 5×Q-Solution (Qiagen), 2.5 μl of 10×PCR Buffer and 1 unit of HotStarTaq *Plus *DNA polymerase (Qiagen) at the Gene Amp^@ ^PCR System 9700 (Applied Biosystem). The PCR cycling profile was as follows: initial denaturation at 98°C for 5 minutes; 35 cycles at 98°C for 45 seconds, 70°C for 45 seconds, and 72°C for 2 minutes, and a final extension at 72°C for 10 minutes. PCR products were purified using DyeEx™ 96 plates (Qiagen) before fragment analysis and sizing by capillary electrophoresis using an automatic sequencer (MegaBACE™ 1000 – GE HealthCareAmersham). Allele sizes were determined using Genescan ROX-550 or ROX-900 internal standard markers (Amersham Bioscience). In a 96-well full-skirted microtitre plate, 5 μl of purified PCR product was added to 5 μl of ROX marker and heated to 95°C for 30 seconds. The 96-well microtitre plate was then loaded onto the MegaBACE DNA sequencer and run according to the manufacturer's instructions. The collected data were analysed using MegaBACE Fragment profiler version 1.2 (Amersham Bioscience). Each run included a number of control samples of lengths 10, 23, 29, 30, 52 and 74 repeats determined by sequencing in-house or obtained from Coriell Cell Repositories[5].

DNA sequencing was performed using the Big Dye ve3.1 Terminator cycle sequencing kit (Applied Biosystems), and betaine (trimethylglycine) (Sigma).

Reactions were performed in a 20 μl reaction volume containing of 1 μl Big Dye ve3.1 Ready Mix, 3.5 μl 5× BDT dilution buffer, 0.5 μl of primer *c *or *f *(10 μM/μl), 10–12 ng of purified PCR product (High Pure PCR Product Purification Kit, Roche), and 4 μl of 5 M betaine dH_2_O at PCR tube. The reaction was performed for 1 cycle at 98°C for 3 minutes followed by 25 cycles of 98°C for 30 seconds and 60°C for 4 minutes. The samples were precipitated by adding 75 μl of fresh 0.2 mM MgSO_4 _to each tube at room temperature and mixed thoroughly by vortexing, then allowed to sit at room temperature for a minimum of 15 minutes. The tubes were centrifuged at 13,000 rpm in a bench top centrifuge for 15 minutes and supernatants discarded. The pellets were air-dried for 5 minutes and sequenced.

## Results

### PCR Analysis

**Figure 1:**
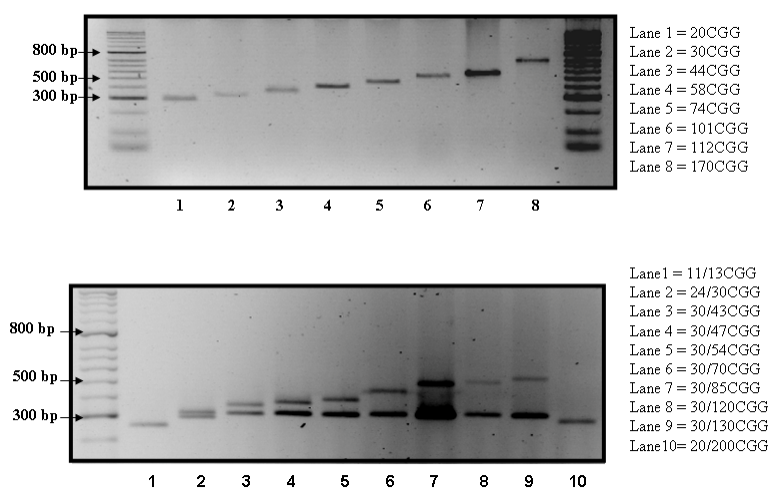
**This figure shows the expanded range of allele sizes (in triplet repeats) detectable with the newly designed assay using ethidium bromide detection.** Allele size was determined by standard curve analysis.

The expanded range of allele sizes in both males and females detectable using the improved assay is shown in Fig 1. This is at least 170 CGG repeats in males and 130 CGG repeats in females by gel visualisation. This compares to an upper limit of 80 CGG repeats in male and female samples for the previous PCR assay.

Validation of the results obtained using the improved assay has been performed in two ways. The measurements obtained by sequencing of 52 samples ranging from 10 to 112 repeats (the upper limit of sequencing these CG rich regions) were compared with measurements (in triplets) of CGG repeat length using the improved PCR assay. There was a linear relationship, Y (sequenced repeat number) = X (assay repeat number) + 2.4, and a correlation coefficient r^2 ^= 0.999. This correlation was used to correct all improved assay measurement by addition of 2 triplet repeats (Y-axis intercept, 2.4 repeats). Secondly, the results obtained with the improved assay correlated very highly with those of the old assay across the range of amplification available with the latter (r^2 ^= 0.997, Y (improved assay) = 0.94× (old assay) – 0.58). Taking these two correlations together, results obtained with the old assay are 3 triplet repeats higher (Y-axis intercepts of 2.4 + 0.58) than those of the improved assay using the sequenced repeat data as the standard.

There was no variation associated with different internal sequence structures in terms of intervening AGG triplets which numbered from none to three per allele for alleles of 110–112 repeats.

**Figure 2:**
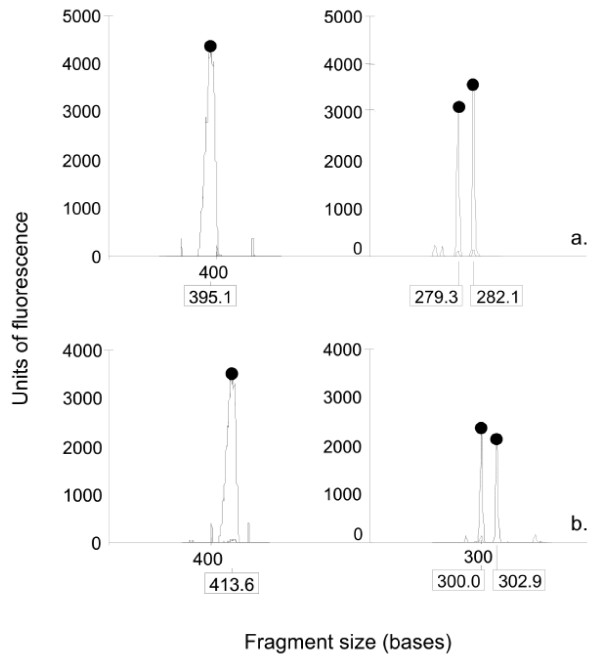
Comparison of old (left) and new (right) assays in two patients illustrating the increased sensitivity when analysing females whose alleles differ by a single CGG triplet repeat.

The profiles obtained on capillary electrophoresis with the new assay showed sharp, discrete peaks (Fig 2a and 2b). This was particularly evident with longer alleles where comparable peaks from the old assay typically showed broader, stuttered profiles. These sharpened peaks provided significantly improved resolution. Re-analysis of 83 female samples with the new assay, which were previously reported as single allele homozygotes, showed that 46 (53%) were actually heterozygotes with two alleles differing by a single triplet repeat (Fig 2a and 2b). All apparent homozygous females contained alleles in the ranges 20 to 23 repeats and 29 to 31 repeats where the most frequent alleles are clustered [6-8].

**Figure 3:**
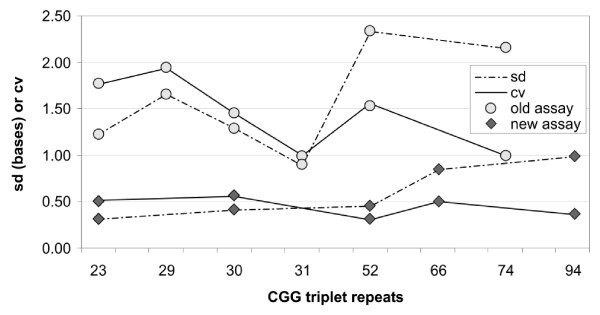
Comparison of the sensitivity between the old and the newly designed assays.

The precision and uncertainty of measurement of the old and new assays was compared by analysing the results of serial repeats of control samples used in diagnostic runs. Controls with sizes of 20, 23, 30, 52, 66, 74 and 94 CGG repeats were used. Standard deviations and coefficients of variation were lower across the range for the new assay compared with the old assay (Fig 3). The general practice of calculating 95% confidence limits for pathology measurements indicates an uncertainty range of +/- 1.96 CVs about the measured value. As a maximum CV of 1.94 was found at 29 repeats using the old assay and 0.54 at 30 repeats for the new assay, the uncertainly of measurement for the two assays is less than +/- 3.9% and +/- 1.1% respectively across the range 23 to 74 CGG repeats.

## Discussion

Fragile X syndrome is one of the most common genetic disorders with a carrier frequency of approximately 1 in 800 males and 1 in 260 female in the general population [8-10]. Its morbidity, relatively high frequency, the benefits of early diagnosis and the crucial importance of identifying and offering prenatal diagnosis to carriers presents a strong case for widespread screening of the mentally retarded population and provision of prenatal diagnosis. Furthermore, the recent association of premutations with two different disorders, FXTAS and POF [8,11-13], underscores the importance of testing for *FMR1 *CGG triplet repeat expansions. However, widespread testing has been impeded by the lack of a single simple, cost-effective test. Although detection of a normal result in males excludes *FMR1 *premutations or full mutations, approximately 40% of females require a further time-consuming, complex and expensive Southern blot test which distinguishes homozygotes from carriers of larger alleles which PCR testing fails to detect.

The aim of this study was to develop an improved PCR assay to delineate cryptic heterozygous females and to extend the repeat size range of alleles detected. This would reduce the number of follow-up Southern blot tests required, improving result turnaround times and reduce costs of additional testing.

PCR amplification of tracts of CGG repeats greater than 70 repeats is problematic due to the high GC-rich content which promotes formation of alternative DNA structures [14] and the creation of polymerase pause sites which introduces errors during replication [15]. A number of PCR techniques have already been described which use diverse combinations of DNA polymerase and inhibiting secondary structure co-solvents such as dimethyl sulfoxide (DMSO) and betaine [8,14,16-18].

Our approach was to investigate using newer DNA polymerases with improved fidelity and capability for amplifying CG-rich sequences and to utilize primers that locate closer to the CGG repeat region. HotStarTaq *Plus *DNA polymerase was chosen to prevent extension of non-specifically annealed primers and primer-dimers complexes formed at low temperatures during PCR setup and the initial PCR cycle. By changing to HotStarTaq *Plus *polymerase with Q- Solution and primers *c *and *f *[4], the upper range of amplifiable allele sizes was increased by at least 100% and 60% for male and female samples respectively (Fig 1). Typically for amplification of any repeat sequence, the PCR products from the old assay showed stuttered peaks when separated by capillary electrophoresis, especially for alleles of greater than 50 repeats. The selection of new primers (*c *and *f*) [4] has reduced the length of the PCR product for a 30 repeat allele from 419 base pairs for the old primers to 300 base pairs for the new primers. This has increased the efficiency of the PCR and theoretically maximizes the potential for separating alleles that differ by a single triplet repeat. Somewhat unexpectedly, the amplified products from the improved assay showed significantly sharper peaks with consequent improvement in precision of measurement. Identification of the exact source of this improvement was attempted by swapping primers and enzymes between the old and improved assays protocols with no clear answer, indicating that most likely a combination of using the optimized conditions of hot-start enzyme with its proprietary buffer and the new primers is responsible. Alleles up to 112 repeats could be sequenced and these were used to validate the assay's precision. Given this level of correlation (r^2 ^= 0.999) and its linearity y = x + 2.4, the improved assay's uncertainty of measurement, defined as +/-1.96 CVs, varies from +/- < 1 triplet repeat at 20 repeats to +/- 3 CGG triplet repeats at 170 repeats, the upper limit of the new assay's range. Using the sequenced allele data as a standard across the range of alleles from 10 up to 112 repeats, the upper limit of sequencing this CG-rich template, the correlation found between the old and new assays infers that the old assay's measurements are about 1 triplet repeat higher across the range of 10–40 repeats.

Validation of *FMR1 *CGG triplet repeat assays used in diagnostic laboratories is challenging due to the lack of reliable standards. Sequenced standards are not widely used and even here the extreme CG-rich nature of the triplet repeats makes accurate sequencing difficult. The same applies to cloning of alleles for use as standards or use of commercially available lymphoblastoid lines. Although errors of the order of 1–3 repeats would have no bearing on the diagnosis of FXS they would be significant for the detection of intermediate and small premutation alleles, relevant to FXTAS and POF. There is an obvious need for provision of a set of standards to be used internationally and this is in process of being addressed [19].

The higher precision of the improved assay was used to check the status of 83 samples which had previously given a mono-allelic result using the old assay. All had been further analysed by Southern blot and premutations or full mutations were excluded in all. The assay showed that 46 (53%) were in fact biallelic with alleles differing by one CGG triplet. As homozygous results were found in 53% of female samples using the old assay, this finding significantly reduces the proportion requiring follow-up Southern blot testing.

The other important improvement provided by use of the new assay is to identify alleles with the potential to be unstable when transmitted to offspring. These are grey zone alleles (45 < CGG repeats < 54) and small premutation alleles (55 < CGG repeats < 100 CGG)[20]. Additionally, precise measurement of alleles of 59 repeats or more is important as 59 repeats is the smallest recorded allele which has expanded to a full mutation on transmission[21].

## Conclusion

The new assay offers a significant improvement in resolution and precision for measurement of alleles in the range of 6 to 170 CGG repeats can be distinguished. Use of this assay provides turnaround and cost advantages for diagnostic laboratories performing high numbers of Fragile X syndrome tests. Importantly, the assay reduces by half the number of follow-up Southern blot tests which are otherwise required to exclude premutations or full mutations in females with apparent homozygous alleles. The high precision provided will also be of value in ongoing research focussing on the biological interrelationships and clinical significance of normal, gray zone and small FMR1 mutations.

## Competing interests

The author(s) declare that they have no competing interests.

## Authors' contributions

MK and HS participated in the study concept, design, analysis and acquisition of data. MK and TB performed the preparation and molecular analysis of the samples. MK and HS drafted the manuscript and performed the statistical analysis. MK, HS, TB and PK participated in drafting and critical revision of the manuscript HS and PK participated in the studies supervision. All authors read and approved the final manuscript.
